# Sulfonated graphene oxide/MnFe_2_O_4_ magnetic nanocomposite for effective 5-hydroxymethylfurfural etherification in biofuel production

**DOI:** 10.3389/fchem.2026.1905987

**Published:** 2026-07-22

**Authors:** Almuhannad S. Alorfi, Nidal H. Abu-Hamdeh

**Affiliations:** 1 Department of Information Systems, Faculty of Computing and Information Technology, King Abdulaziz University, Jeddah, Saudi Arabia; 2 Department of Mechanical Engineering, Center of Research Excellence in Renewable Energy and Power Systems/Energy Efficiency Group, Faculty of Engineering, King Abdulaziz University, Jeddah, Saudi Arabia

**Keywords:** 5-ethoxymethylfurfural, 5-hydroxymethylfurfural, acid catalysis, graphene oxide, MnFe_2_O_4_ nanoparticles

## Abstract

Herein, a solid acid catalyst, SO_3_H-GO/MnFe_2_O_4_, was synthesized through a three-step process, including the preparation of graphene oxide (GO), deposition of MnFe_2_O_4_ nanoparticles onto GO, and subsequent sulfonation with chlorosulfonic acid. The synthesized catalyst was completely analyzed by numerous techniques, including FT-IR, TEM, XRD, EDX, VSM, and acid-base titration, approving the successful incorporation of MnFe_2_O_4_ nanoparticles and -SO_3_H groups onto GO. The activity of prepared SO_3_H-GO/MnFe_2_O_4_ was determined in the etherification of 5-hydroxymethylfurfural (HMF) to 5-ethoxymethylfurfural (EMF). The reaction factors were optimized and the optimum condition was obtained at 120 °C, 30 mg of catalyst dosage, and time of 6 h, providing high EMF yield (93.2%), and selectivity (96%). The prepared particles were easily isolated from the mixture by a magnet and showed excellent recyclability, retaining most of its activity over four consecutive cycles. Moreover, the catalyst was also efficient in the direct conversion of biomass-derived sugars (fructose, glucose, and sucrose) to EMF.

## 1 Introduction

The reduction of fossil fuels and the environmental challenges associated with their widespread utilization have driven intensive global efforts toward the use of renewable and sustainable energy alternatives (Baruah et al., 2025). Lignocellulosic biomass is now regarded as one of the most promising feedstocks for the synthesis of biofuels and valuable chemicals (Aydan et al., 2026; Gujar et al., 2025). Unlike first-generation resources like sugarcane or corn, lignocellulosic biomass does not compete with the food chain and can be obtained from agricultural residues, industrial waste streams, and forestry byproducts (Shabih et al., 2018). Transforming this complex renewable material into easily transportable and energy-dense liquid fuels needs efficient catalytic systems able to convert key biomass-derived platform molecules. Among these, 5-hydroxymethylfurfural (HMF) has received significant attention owing to its multifunctional structure containing both hydroxyl and carbonyl groups, which makes it an outstanding intermediate for the synthesis of a wide range of fine chemicals, fuels, and polymers (Amin et al., 2025; Li J. et al., 2025).

One of the most attractive transformations of HMF is its etherification to 5-ethoxymethylfurfural (EMF). EMF has been regarded as a next-generation biofuel candidate thanks to its high energy density (∼30 MJ·L^-1^), low toxicity, and excellent blending compatibility with diesel fuels (Chen et al., 2020; Jeong, 2026). EMF can be utilized directly as a fuel additive or as an intermediate for advanced biofuel production (Torres-Olea et al., 2025). The conversion of HMF to EMF usually requires acid-catalyzed etherification in ethanol media (Zuo et al., 2023). Accordingly, the selection of an efficient and sustainable acid catalyst is essential for attaining high EMF yield.

Traditional homogeneous acids such as sulfuric and p-toluenesulfonic acid exhibit high activity in HMF etherification but suffer from serious problems including corrosion, difficulty in catalyst separation and recovery, and the generation of large volumes of acidic waste (Liu and Wang, 2018). To overcome these problems, research has shifted toward the use of heterogeneous acid catalysts, which offer various advantages like easy separation, reusability, reduced environmental impact, and improved operational simplicity (Hafizi et al., 2020; Wang and Chen, 2016). Solid acids with strong Brønsted acidity, mainly sulfonated materials, have shown outstanding catalytic activity for etherification of HMF (Yu D. et al., 2021). Nevertheless, for solid acids to function efficiently in HMF etherification, they must provide high acid site density, good stability in polar solvents like ethanol, and enough surface area to ensure accessibility of active sites.

Carbon-based materials, particularly graphene oxide (GO), have appeared as attractive supports for the design of heterogeneous catalysts. GO possesses a high surface area and a high density of oxygen functional groups which improve dispersion in polar media and allow strong interactions with metal ions or nanoparticles (Kandathil et al., 2020; Wang et al., 2019). GO also reveals good thermal stability and can be easily functionalized with acidic groups, making it a suitable support for preparing heterogeneous acid catalysts (Miranda et al., 2019). Though, despite these advantages, the separation of graphene oxide from reaction mixtures is difficult, which render the practical application of GO-based catalysts. On the other hand, conventional filtration or centrifugation is often time-consuming and inefficient, which limits the recyclability and practical usefulness of GO-based catalysts (Faruque et al., 2021).

To overcome the above problem, magnetic nanoparticles have been successfully incorporated into GO-based catalysts (Tahmasbi et al., 2024). Among magnetic ferrites, manganese ferrite (MnFe_2_O_4_) is prominent due to its strong magnetic behavior and high thermal stability. Magnetic properties of MnFe_2_O_4_ nanoparticles provide rapid and simple separation from reaction mixtures using an external magnet (Liew et al., 2019). Combining GO with MnFe_2_O_4_ yields a GO/MnFe_2_O_4_ nanocomposite with synergistic advantages compared with the pure components that make it highly attractive supports for designing multifunctional catalysts (Bai et al., 2021; Li W. et al., 2025; Yu Y. et al., 2021).

Although variuos magnetic sulfonated carbon-based catalysts have been reported for HMF etherification, numerous challenges remain. Many previously reported catalysts used Fe_3_O_4_ as the magnetic component, which may suffer from limited chemical stability under acidic reaction conditions or reduced magnetic performance after repeated use. Furthermore, attaining a balance between high Brønsted acid-site density, catalyst stability, efficient magnetic recovery, and accessibility of active sites remains an important challenge. Thus, developing robust magnetic supports capable of maintaining high catalytic activity whereas enabling facile catalyst recycling is still of considerable interest.

In this study, we developed a magnetically separable solid acid catalyst (SO_3_H-GO/MnFe_2_O_4_) specifically designed for efficient HMF etherification and sugar-to-EMF conversion. Unlike previously reported magnetic sulfonated carbon catalysts, our catalyst combined high acid site density and rapid magnetic recovery, offering both practical recyclability and enhanced catalytic performance in converting biomass-derived substrates into EMF. Go was first synthesized and subsequently decorated with MnFe_2_O_4_ nanoparticles to overcome the separation limitations associated with the pristine GO and to introduce magnetic properties for easy recyclability. The resulting nanocomposite was then functionalized with chlorosulfonic acid to produce strong Brønsted acid sites essential for HMF etherification. After fully characterization, it was applied as a heterogenous acid catalyst for the etherification of HMF to EMF.

## 2 Experimental

### 2.1 General

All chemicals, reagents, and solvents used in this work were purchased from Sigma-Aldrich or Merck. They were of analytical grade and were used without further purification. Deionized water was used throughout all synthesis and washing steps. The prepared materials were characterized using a range of physicochemical techniques. Detailed descriptions of the characterization methods and instrumental conditions are given in the Supplementary Material.

### 2.2 Synthesis of graphene oxide (GO)

GO was prepared from graphite powder using a modified Hummers’ process (Zamani and Tadjarodi, 2024). Concisely, graphite powder (0.500 g) and sodium nitrate (0.375 g) were mixed in concentrated sulfuric acid (40 mL) under continuous stirring in an ice bath. Potassium permanganate (2.250 g) was gradually added while preserving the temperature below 10 °C. The mixture was stirred for 5 days. Then, 70 mL of aqueous solution of 5% w/w H_2_SO_4_ was added, followed by further heating to 98 °C for 1 h. The reaction was finished by adding 2 mL of 30% w/w hydrogen peroxide, followed by stirring for 2 h. The resultant GO was rinsed repeatedly with dilute HCl solution and deionized water until neutral pH, then dried at 60 °C in a vacuum oven for 12 h.

### 2.3 Synthesis of GO/MnFe_2_O_4_ nanocomposite

MnFe_2_O_4_ nanoparticles were deposited onto the GO nanosheets through a co-precipitation method (Fakhri-Mirzanagh et al., 2024). GO (0.25 g) was dispersed in deionized water (50 mL) through ultrasonication to form a stable suspension. Afterward, MnCl_2_.4H_2_O (0.63 g) and FeCl_3_.6H_2_O (0.68 g) were dissolved in DI water (10 mL) and added to the GO suspension under vigorous stirring. The mixture was heated to 80 °C, and 30 mL of NaOH (3M) was slowly introduced, facilitating the formation of MnFe_2_O_4_ nanoparticles on the GO surface. The mixture was stirred for an additional 1 h at 80 °C. The obtained solid (GO/MnFe_2_O_4_) was magnetically separated, washed repeatedly with water and ethanol, and dried at 80 °C under vacuum.

### 2.4 Synthesis of SO_3_H-GO/MnFe_2_O_4_

**SCHEME 1:**
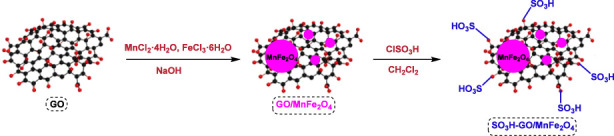
Preparation process of SO_3_H-GO/MnFe_2_O_4_.

GO/MnFe_2_O_4_ (1 g) was dispersed in dry dichloromethane (30 mL) with continuous stirring. Chlorosulfonic acid (10 mL) was added dropwise to the suspension during 1 h. The reaction mixture was stirred at 0 °C for 12 h. The solid particles (SO_3_H-GO/MnFe_2_O_4_) were then separated, rinsed by cold ethanol and deionized water to remove unreacted reagents, and dried at 60 °C in a vacuum oven for 12 h. Preparation process of the catalyst is presented in Scheme 1.

### 2.5 Measuring the acid content

The acidity of both the fresh and used catalysts was evaluated by standard titration. For the analysis, 5 mg of each compound was mixed with 10 mL of saturated sodium chloride solution and stirred for 6 h. Afterward, the catalyst was isolated and the filtrate was titrated against 0.01 M NaOH using phenolphthalein as the endpoint indicator.

### 2.6 Catalytic etherification of HMF to EMF

The catalytic etherification of HMF with ethanol was conducted in a 50 mL Teflon stainless-steel autoclave. HMF (1 mmol), ethanol (5 mL), and a known amount of SO_3_H-GO/MnFe_2_O_4_ as catalyst were added to the autoclave and heated to the desired reaction temperature under continuous stirring for a specified time. Once the reaction is over, the catalyst was magnetically isolated from the mixture, and the reaction mixture was filtered and analyzed by high-performance liquid chromatography (Agilent 1260) with a C18 reverse-phase column (200 mm × 4.6 mm, 5 μm). A 0.1 wt% aqueous solution of acetic acid as well as acetonitrile (V: V = 85:15) was used as the mobile phase at 30 °C with a flow rate of 0.5 mL/min. HMF conversion, EMF yield, and EMF selectivity were determined based on these equations:

$$\text{HMF Conversion percentage }\left(\%\right)=\left[\text{moles of reacted HMF }/\text{ moles of initial HMF}\right]\times 100\%$$

$$\text{EMF yield }\left(\%\right)=\left[\text{moles of EMF formed }/\text{ moles of initial HMF}\right]\times 100\%$$

$$\text{EMF selectivity }\left(\%\right)=\left[\text{EMF yield }/\text{ HMF conversion}\right]\times 100\%$$

### 2.7 Catalyst reusability

To assess the reusability of SO_3_H-GO/MnFe_2_O_4_, the recovered catalyst after each run was rinsed thoroughly with ethanol and dried at 80 °C before reuse. The etherification reaction was repeated under identical conditions for four consecutive cycles to evaluate catalytic stability and activity.

## 3 Results and discussion

**FIGURE 1:**
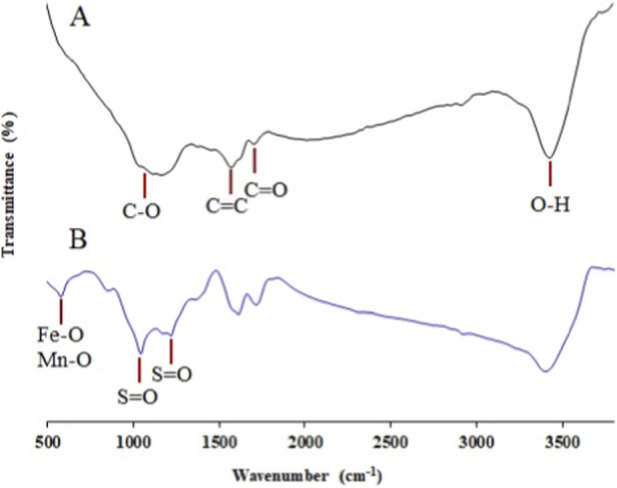
FT-IR spectra of GO **(A)** and SO_3_H-GO/MnFe_2_O_4_ **(B)**.

Fourier-transform infrared (FT-IR) spectroscopy was used to approve the successful formation of MnFe_2_O_4_ nanoparticles on GO and the subsequent sulfonation. FT-IR spectra of the pristine GO and SO_3_H-GO/MnFe_2_O_4_ are presented in Figure 1. The FT-IR spectrum of the pristine GO exhibited characteristic peaks around 3,425 cm^-1^ (O-H stretching), 1,703 cm^-1^ (C=O stretching), 1,574 cm^-1^ (C=C vibration), and 1,039 cm^-1^ (C-O stretching), consistent with the existence of oxygenated functional groups (Zourou et al., 2023). In the FT-IR spectrum of SO_3_H-GO/MnFe_2_O_4_, a new peak appeared around 577 cm^-1^, attributed to the Fe-O and Mn-O vibrations in the spinel structure, endorsing the effective formation of MnFe_2_O_4_ nanoparticles on the surface of GO (Xu et al., 2018). In addition, other two peaks at 1,047 cm^-1^ and 1,158 cm^-1^, ascribed to the S=O stretching of -SO_3_H groups, indicating the effective functionalization of the nanocomposite with Brønsted acid sites (Rezaei et al., 2020). These results collectively suggested the successful integration of GO, MnFe_2_O_4_, and sulfonic acid groups in the catalyst.

**FIGURE 2:**
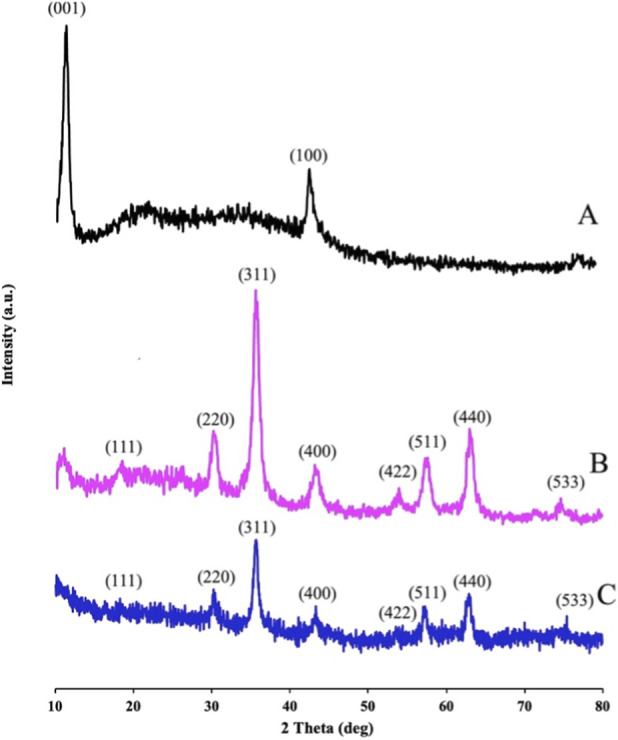
XRD patterns of GO **(A)** GO/MnFe_2_O_4_ **(B)** and SO_3_H-GO/MnFe_2_O_4_ **(C)**.

The X-ray diffraction (XRD) patterns of GO, GO/MnFe_2_O_4_, and SO_3_H-GO/MnFe_2_O_4_ were recorded to evaluate their crystallinity and phase composition (Figure 2). GO revealed two diffraction peaks at 2θ = 11.2° and 42.8°, characteristic of the (001) and (100) crystal planes of oxidized graphite, respectively (Zourou et al., 2023). After loading MnFe_2_O_4_ onto GO, new diffraction peaks appeared at 2θ = 18.6°, 30.4°, 35.7°, 43.6°, 54.0°, 57.7°, 63.1°, and 74.8°, which were ascribed to the (111), (220), (311), (400), (422), (511), (440), and (533) crystal planes of spinel MnFe_2_O_4_ (Xu et al., 2018). However, the intensities of the GO-related peaks markedly decreased in the XRD pattern of GO/MnFe_2_O_4_, demonstrating coverage of GO layers by the formed MnFe_2_O_4_ nanoparticles. After sulfonation, SO_3_H-GO/MnFe_2_O_4_ retained the diffraction peaks of MnFe_2_O_4_, while with slightly reduced intensities, which can be ascribed to surface modification and possible dispersion effects induced by -SO_3_H functionalization. Notably, the characteristic peak of GO was no longer observed in the XRD pattern of SO_3_H-GO/MnFe_2_O_4_, indicating successful chemical modification of the GO sheets and further structural exfoliation or masking by -SO_3_H functional groups. These observations collectively approved the sequential formation and functionalization of GO/MnFe_2_O_4_ nanocomposites.

The total acidic capacity of SO_3_H-GO/MnFe_2_O_4_ was measured by acid-base titration. Acid-base titration exhibited that SO_3_H-GO/MnFe_2_O_4_ had a total acid density of 1.78 mmol g^-1^, which was markedly higher than that of the unsulfonated GO/MnFe_2_O_4_ (0.34 mmol g^-1^). This considerable increase confirmed grafting of -SO_3_H onto the surface of GO and indicated the enhancement of Brønsted acidity attained through the sulfonation treatment.

**FIGURE 3:**
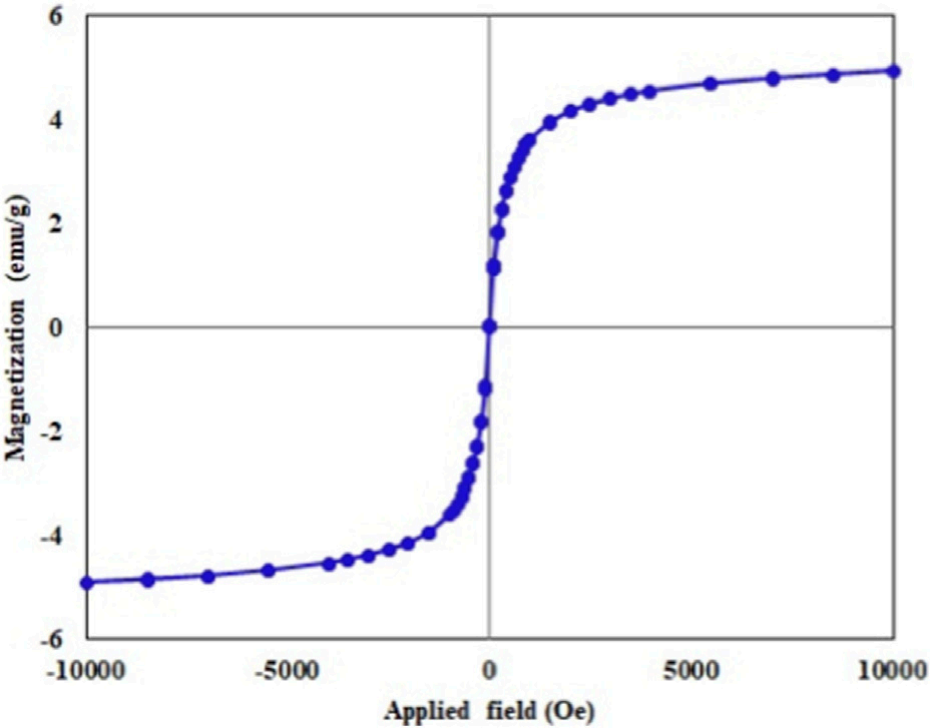
VSM curve SO_3_H-GO/MnFe_2_O_4_.

The magnetic feature of SO_3_H-GO/MnFe_2_O_4_ was investigated using vibrating sample magnetometry (VSM). As shown in Figure 3, the saturation magnetization of SO_3_H-GO/MnFe_2_O_4_ was found to be 4.9 emu/g. This clearly confirmed that SO_3_H-GO/MnFe_2_O_4_ retained sufficient magnetization for easy magnetic separation, making it a magnetically recoverable catalyst.

**FIGURE 4:**
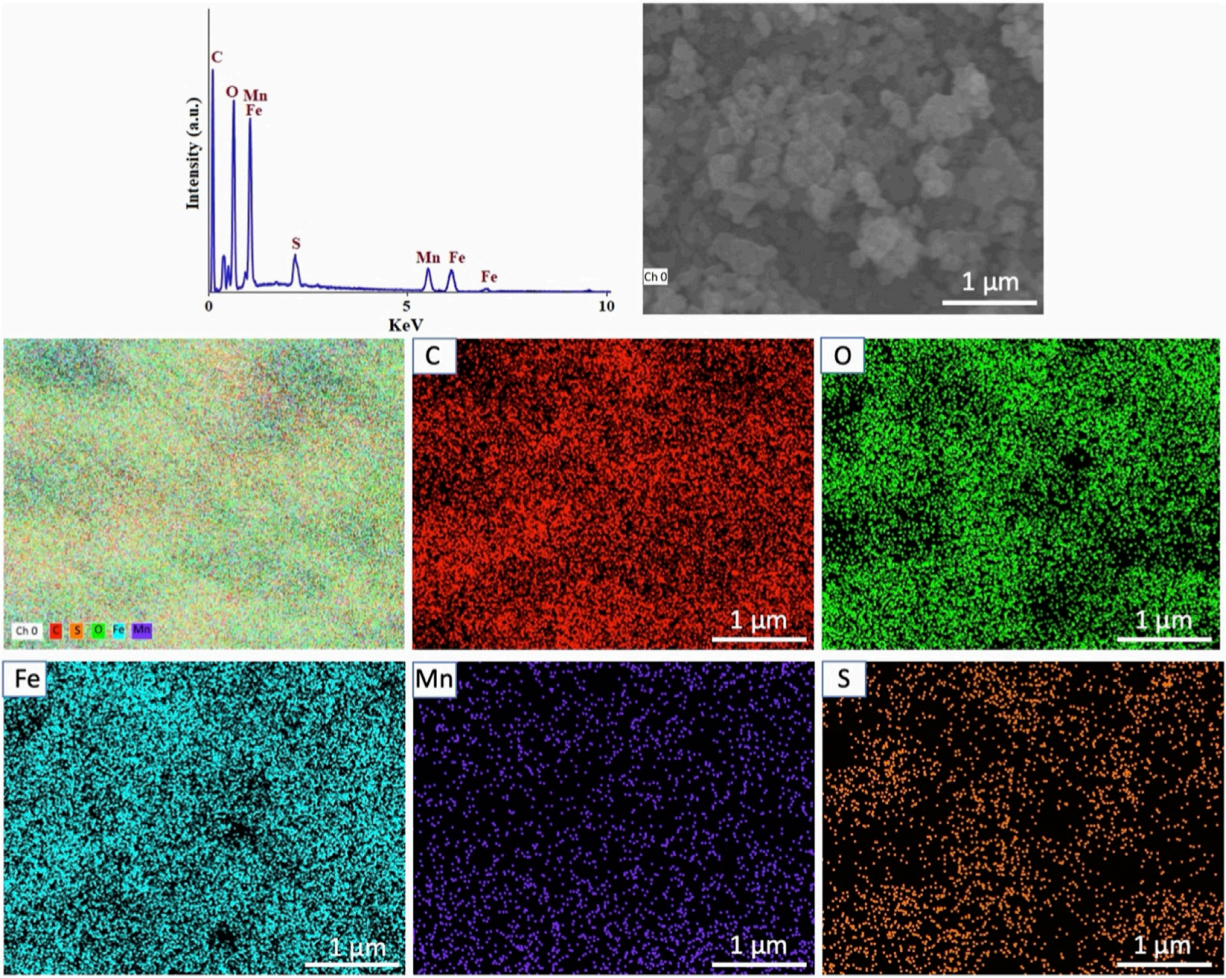
EDX pattern and corresponding elemental mapping images of SO_3_H-GO/MnFe_2_O_4_.

Energy dispersive X-ray spectroscopy (EDX) analysis of SO_3_H-GO/MnFe_2_O_4_ (Figure 4) established the existence of C, O, Mn, Fe, and S elements, representing successful incorporation of MnFe_2_O_4_ and sulfonic acid groups onto GO. Elemental mapping illustration further revealed uniform distribution of C, O, Mn, Fe, and S across the GO surface, confirmed uniform functionalization of the catalyst.

**FIGURE 5:**
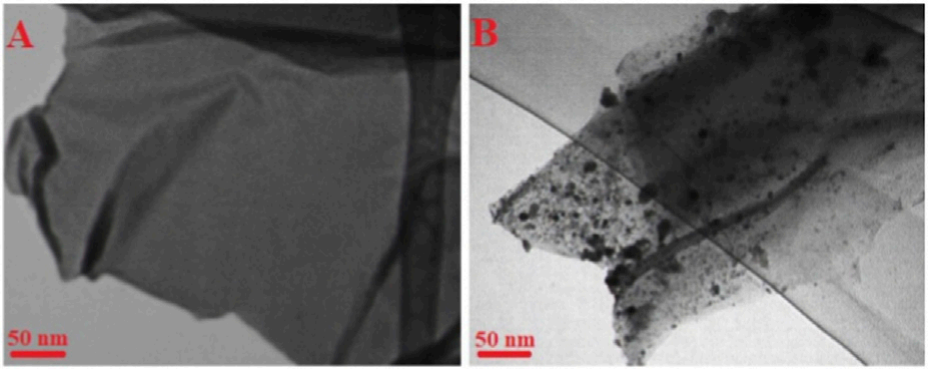
TEM images of GO **(A)**. and SO_3_H-GO/MnFe_2_O_4_ **(B)**.

The morphology of GO and SO_3_H-GO/MnFe_2_O_4_ was investigated by transmission electron microscopy (TEM) analysis (Figure 5). TEM image of the pristine GO exhibited thin, transparent, and wrinkled sheet-like layers with smooth surfaces and no visible particulate deposits, demonstrating successful exfoliation of graphite and the formation of few-layer GO. In contrast, the TEM image of SO_3_H-GO/MnFe_2_O_4_ showed GO sheets uniformly decorated with dark, spherical MnFe_2_O_4_ nanoparticles. The nanoparticles were well-dispersed across the GO surface, indicating effective nucleation and anchoring during the co-precipitation process. Moreover, the sheet-like structure of GO was preserved after nanoparticle deposition and sulfonation.

The catalytic performance of SO_3_H-GO/MnFe_2_O_4_ was examined in the conversion of HMF to EMF utilizing ethanol as both solvent and reactant. After confirming the successful sulfonation of GO/MnFe_2_O_4_ through comprehensive characterization, the catalyst was assessed under various reaction conditions to determine its optimal performance. For all experiments, three parameters were checked: HMF conversion, EMF yield, and EMF selectivity.

**FIGURE 6:**
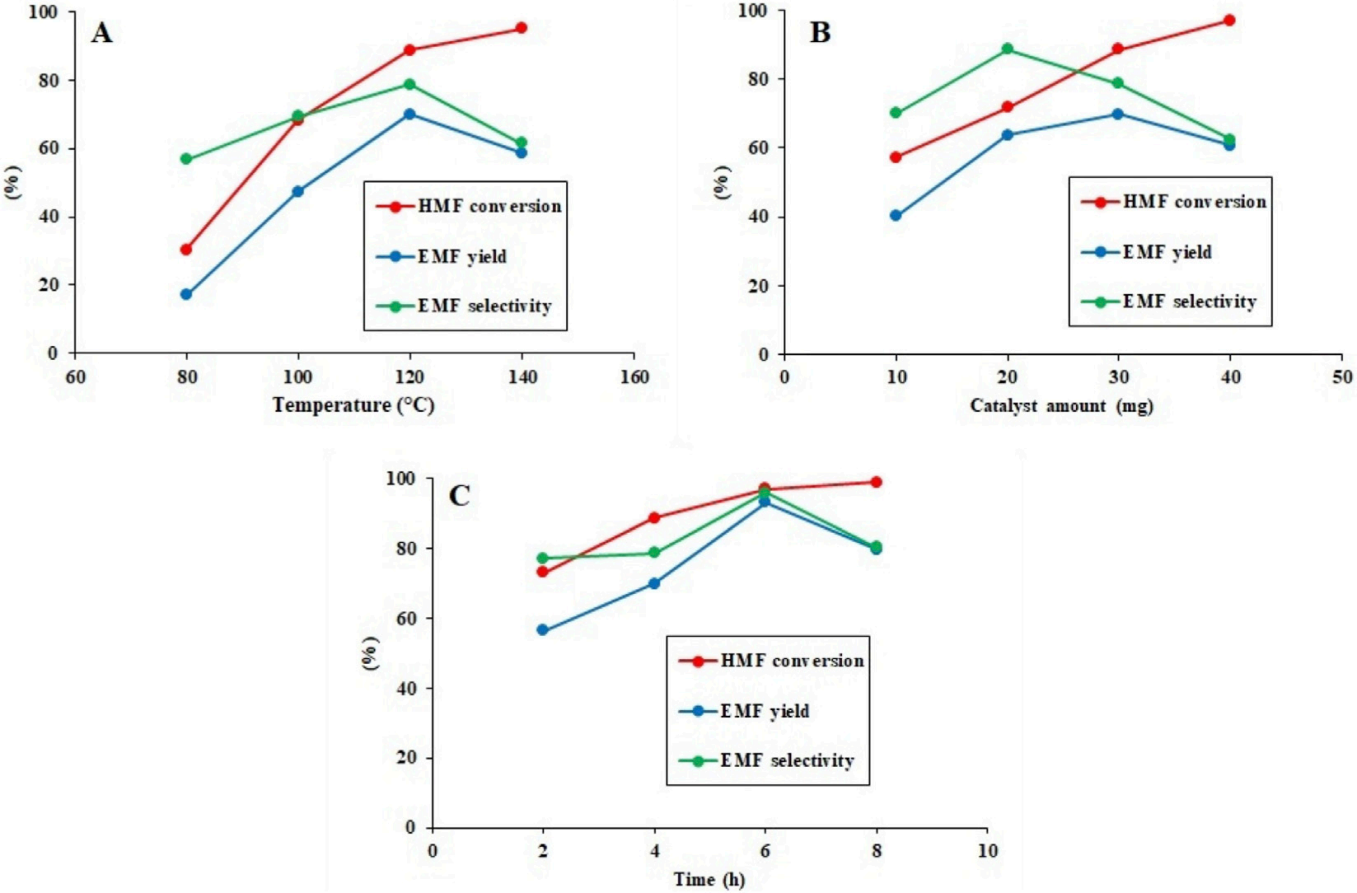
Etherification of HMF over SO_3_H-GO/MnFe_2_O_4_. **(A)** Influence of temperature (reaction conditions: HMF (1 mmol), ethanol (5 mL), SO_3_H-GO/MnFe_2_O_4_ (30 mg), 4 h); **(B)** influence of catalyst dosage (reaction conditions: HMF (1 mmol), ethanol (5 mL), 120 °C, 4 h); **(C)** influence of time (reaction conditions: HMF (1 mmol), ethanol (5 mL), SO_3_H-GO/MnFe_2_O_4_ (30 mg), 120 °C).

The reaction was performed at four temperatures (80, 100, 120, and 140 °C) under otherwise identical conditions (30 mg catalyst, 4 h). As presented in Figure 6A, the reaction temperature strongly influenced the catalytic performance. At 80 °C, HMF conversion was low owing to the insufficient activation of the Brønsted acid sites, which resulted in a poor EMF yield and correspondingly low EMF selectivity. Enhancing the temperature to 100 °C improved the catalytic performance, providing higher HMF conversion and moderate EMF yield. A significant enhancement was observed at 120 °C, where the HMF conversion reached its highest value (88.7%), accompanied by the maximum EMF yield (69.9%) and EMF selectivity (78.8%). However, at 140 °C, both EMF yield and selectivity slightly reduced despite high HMF conversion. This decline was ascribed to secondary reactions like humin formation and EMF degradation, which consumed the product at high temperatures (Hafizi et al., 2022). Consequently, 120 °C was selected as the optimal temperature.

To investigate the effect of catalyst amount, the reaction was conducted using 10, 20, 30, and 40 mg of the catalyst at 120 °C for 4 h (Figure 6B). When using 10 mg of the catalyst, HMF conversion was limited, resulting in small reaction yield. Increasing the catalyst to 20 mg improved HMF conversion and EMF yield owing to the accessibility of more active acid sites. The highest HMF conversion (88.7%), EMF yield (69.9%), and EMF selectivity (78.8%) were attained with 30 mg of the catalyst, demonstrating that sufficient sulfonic acid groups were present to promote the reaction efficiently. Further enhancing the catalyst loading to 40 mg led to a slight decline in EMF yield and selectivity. This can be ascribed to possible mass-transfer limitations or excessive adsorption of substrates on the overly abundant active sites, which can reduce effective interaction between HMF and ethanol. Consequently, 30 mg was selected as the optimal catalyst loading.

The reaction time was varied from 2 to 8 h (120 °C, 30 mg catalyst) and the results are revealed in Figure 6C. At 2 h, both HMF conversion and EMF yield were low, representing incomplete conversion of HMF. After 4 h, HMF conversion increased considerably, accompanied by improved EMF yield and selectivity. The maximum catalytic activity was attained at 6 h, where HMF conversion (97.1%) reached its peak and EMF yield (93.2%) and selectivity (96.0%) were the highest. Beyond this point, prolonging the reaction time to 8 h resulted in a slight drop in EMF yield and selectivity, while HMF conversion remained high. This suggested that longer reaction times favored the formation of byproducts or partial EMF decomposition (Hafizi et al., 2022). Hence, 6 h was identified as the optimal reaction time.

To reveal the individual roles of each component in SO_3_H-GO/MnFe_2_O_4_, control experiments were performed using GO, MnFe_2_O_4_, and GO/MnFe_2_O_4_ under the optimized reaction conditions for HMF etherification. None of these materials demonstrated significant catalytic performance, confirming that the -SO_3_H Brønsted acid groups were solely responsible for the observed conversion of HMF to EMF. The presence of GO and MnFe_2_O_4_ in the catalyst did not directly contribute to the chemical transformation but provided practical advantages by ensuring uniform dispersion of the -SO_3_H groups on GO, maintaining structural integrity, and enabling rapid magnetic separation. These results highlighted that the enhanced catalytic performance of SO_3_H-GO/MnFe_2_O_4_ aroused from the combination of highly accessible Brønsted acid sites with the structural and recoverable benefits conferred by the MnFe_2_O_4_/GO support.

**FIGURE 7:**
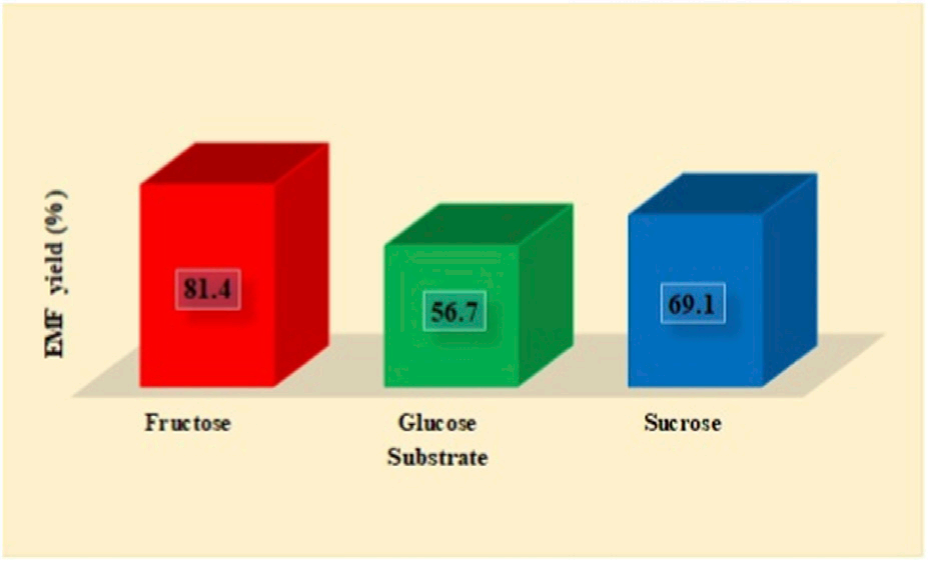
EMF obtained from different sugars over SO_3_H-GO/MnFe_2_O_4_ (reaction conditions: sugar (1 mmol), ethanol (5 mL), SO_3_H-GO/MnFe_2_O_4_ (30 mg), 120 °C, 6 h).

To further assess the versatility of SO_3_H-GO/MnFe_2_O_4_, its performance in the direct conversion of carbohydrates to EMF was studied. Biomass-derived sugars were checked under the optimized reaction conditions previously obtained for HMF etherification (120 °C, 30 mg catalyst, 6 h). The catalytic reaction involves two or more sequential steps: (i) acid-catalyzed dehydration of sugars to HMF, followed by (ii) etherification of HMF with ethanol to form EMF. This multi-step pathway needs catalysts with both strong Brønsted acidity and high stability, making SO_3_H-GO/MnFe_2_O_4_ a suitable candidate. As can be seen in Figure 7, clear differences in reactivity were observed among fructose, glucose, and sucrose. Fructose showed the highest EMF yield, which can be ascribed to its direct and relatively fast acid-catalyzed dehydration to HMF, a well-established and kinetically favorable pathway under Brønsted acidic conditions. Once HMF was formed, it was rapidly transformed to EMF over the sulfonic acid sites, demonstrating that both steps proceed efficiently for fructose-derived intermediates. In contrast, glucose revealed the lowest EMF yield. This behavior was typically associated with its need to undergo an initial isomerization to fructose prior to dehydration to HMF. Under purely Brønsted acidic conditions, this isomerization step was generally slow and was widely recognized in the literature as the main kinetic bottleneck in glucose-to-HMF transformation. Thus, the production rate of HMF was significantly limited, which consequently suppressed EMF production. Sucrose displayed intermediate performance, which was ascribed to its initial acid-catalyzed hydrolysis into glucose and fructose. The fructose fraction was rapidly converted to HMF and subsequently to EMF, though the glucose fraction followed the slower isomerization-dehydration pathway. Consequently, sucrose benefits from the presence of fructose-derived intermediates, leading to higher EMF yield than glucose but slightly lower than fructose alone. The observed results strongly supported a cascade reaction mechanism in which the overall performance was governed by the relative rates of (i) sugar conversion to HMF (hydrolysis/isomerization/dehydration sequence) and (ii) subsequent etherification of HMF to EMF. In particular, the results suggested that for fructose and sucrose, the dehydration step was relatively fast under the applied conditions, while for glucose, the isomerization step acted as the primary rate-limiting factor controlling overall EMF formation. Overall, the catalytic activity followed the order fructose > sucrose > glucose, confirming that SO_3_H-GO/MnFe_2_O_4_ efficiently promoted the sequential hydrolysis-isomerization-dehydration-etherification pathway required for EMF production from biomass-derived carbohydrates.

The practical applicability of a heterogeneous catalyst depends intensely on its stability and reusability. Thus, the recyclability of SO_3_H-GO/MnFe_2_O_4_ was checked in the etherification of HMF to EMF under the optimized reaction conditions (120 °C, 30 mg catalyst, 6 h). After each run, the catalyst was collected via a magnet, thoroughly rinsed with ethanol, dried at 80 °C, and then reused for the next run. As illustrated in Table 1, the catalyst showed outstanding stability over multiple consecutive cycles. After four consecutive cycles, the catalyst reserved the majority of its activity, representing only a minor decline in EMF yield.

**TABLE 1:** Reusability of SO_3_H-GO/MnFe_2_O_4_ in the etherification of HMF under the optimized reaction conditions.

Run	1	2	3	4
EMF yield (%)	93.2	92.9	92.1	91.3

Hot filtration tests was also carried out to further confirm the heterogeneous nature of the catalyst. To this end, the chemical reaction was carried out at the best conditions. The solution was then allowed to continue reacting under identical conditions in the absence of the catalyst. No further imporvement in HMFCA yield was observed after separating the catalyst, demonstrating that no active catalytic species remained in the solution phase. Moreover, ICP analysis of the reaction solution showed no detectable Mn or Fe species, confirming the absence of metal leaching during the reaction. Since the catalytically active sites were the grafted sulfonic acid (-SO_3_H) groups, while the MnFe_2_O_4_ component primarily acted as a magnetic support for facile separation, the slight decrease in catalytic activity upon reuse was most likely due to partial blockage or fouling of accessible acid sites during repeated reaction cycles rather than structural degradation or metal leaching. This result, together with the negligible loss in catalytic activity, confirmed the good stability of SO_3_H-GO/Mn_2_Fe_4_ and the robustness of its surface functionalization during repeated use.

To further evaluate the structural stability of SO_3_H-GO/MnFe_2_O_4_ after four catalytic cycles, acid-base titration, FT-IR, XRD, and TEM analyses were employed to check acid site retention, possible changes in surface functional groups, crystal structure, and morphology, respectively. After four catalytic cycles, the recovered catalyst retained most of its acidity, with only a slight reduction in acid site concentration, endorsing that the majority of the sulfonic acid groups remained intact during the reaction. The FT-IR spectrum of the recycled catalyst showed no considerable differences compared to the fresh sample, demonstrating that all functional groups were preserved after recycling (Supplementary Figure S1). The XRD pattern of the used catalyst remained essentially unchanged, implying that the crystalline structure of the catalyst was well maintained and that no phase transformation occurred under the reaction conditions (Supplementary Figure S2). Additionally, TEM analysis showed that the morphology and dispersion of MnFe_2_O_4_ nanoparticles on the GO support were largely preserved after recycling (Supplementary Figure S3). Though slight nanoparticle aggregation was observed, no significant structural degradation of the support or catalyst framework was evident. These results revealed that SO_3_H-GO/MnFe_2_O_4_ displayed excellent structural stability under the reaction conditions, which was responsible for its good reusability and catalytic durability.

The catalytic performance of SO_3_H-GO/MnFe_2_O_4_ was compared with some of the previously reported heterogeneous catalytic systems in HMF etherification (Table 2). As shown, our catalyst revealed superior EMF yield under relatively mild reaction conditions compared to other solid acid catalysts. Notably, the combination of strong acidic -SO_3_H groups and magnetic MnFe_2_O_4_ nanoparticles not only facilitated high catalytic efficiency but also enabled facile recovery and recyclability, advantages that were less pronounced in previously reported systems. This comparison highlights the practical potential of SO_3_H-GO/MnFe_2_O_4_ for sustainable EMF production.

**TABLE 2:** Comparison of the catalytic performance of SO_3_H-GO/MnFe_2_O_4_ with some reported catalytic systems.

Catalyst	Temperature (°C)	Time (h)	EMF yield (%)	References
SO_3_H-GO/MnFe_2_O_4_	120	6	93.2	[This work]
Fe_3_O_4_@C-SO_3_H	100	12	88.4	Yuan et al. (2015)
SNC-chitin-220	120	6	84.3	Du et al. (2024)
C-SO_3_H	100	6	71	Wang and Chen (2018)
Fe_3_O_4_@SiO_2_-HPW	100	11	76.9	Wang et al. (2013)
K-10 clay-Al	100	8	73.2	Liu et al. (2015)

## 4 Conclusion

In conclusion, SO_3_H-GO/MnFe_2_O_4_ was successfully prepared and characterized as an efficient heterogenous catalyst for biofuel production. The catalyst indicated superior catalytic activity and providing high HMF conversion, EMF yield, and selectivity. Furthermore, the catalyst showed promising activity in the direct conversion of biomass-derived sugars (fructose, glucose, and sucrose) to EMF, with fructose yielding the highest EMF yield. The catalyst exhibited excellent magnetic separability, allowing for efficient recovery after each reaction cycle. After four consecutive catalytic runs, the catalyst maintained high activity, with only a minor reduction in EMF yield. Overall, the results presented in this study highlight the SO_3_H-GO/MnFe_2_O_4_ nanocomposite as a versatile and recyclable catalyst with significant promise for efficient and eco-friendly synthesis of EMF from both HMF and sugars, offering a valued method for the valorization of biomass resources in the context of renewable energy and biofuel synthesis.

## Data Availability

The original contributions presented in the study are included in the article/Supplementary Material, further inquiries can be directed to the corresponding author.
