# Supplementary material for: Sulfonated graphene oxide/MnFe2O4 magnetic nanocomposite for effective 5-hydroxymethylfurfural etherification in biofuel production
Source: Front Chem. 2026 Jul 22;14:1905987. doi: 10.3389/fchem.2026.1905987 (PMC13445599; doi:10.3389/fchem.2026.1905987)
Supplement: Supplementary file 1 [file DataSheet1.docx]

**Supporting Information**

**Sulfonated graphene oxide/MnFe_2_O_4_ magnetic nanocomposite for effective 5-hydroxymethylfurfural etherification in biofuel production**

**Characterizations**

Fourier transform infrared (FT-IR) spectra were recorded on a Nicolet Magna 550 spectrometer using KBr pellet. X-ray diffraction (XRD) patterns were recorded on a X-ray diffraction Bruker D8 Advanced diffractometer. The magnetic property of the catalyst was measured with a vibrating sample magnetometer (VSM, Lakeshore 7300) at room temperature. Energy-dispersive X-ray spectroscopy (EDS) analysis was carried out by a scanning electron microscope (SEM, Hitachi SU8010). Transmission electron microscopy (TEM) images were obtained using a the Jeol JEM-1230 microscope operating at 200 kV.

**Figure S1.** FT-IR spectrum of the recovered catalyst.

**Figure S2.** XRD pattern of the recovered catalyst.

**Figure S3.** TEM image of the recovered catalyst.
